# Evidence-Generated Sockets for Transtibial Prosthetic Limbs Compared With Conventional Computer-Aided Designs: A Multiple-Methods Study From the Patient’s Perspective

**DOI:** 10.2196/69962

**Published:** 2025-08-21

**Authors:** Florence Mbithi, Maggie Donovan-Hall, Jennifer Bramley, Joshua Steer, Charalambos Rossides, Peter Worsley, Chantel Ostler, Cheryl Metcalf, Dominic Hannett, Caroline Ward, Jack Kitchen, Sioned Steventon, Katy McIntosh, Shigong Guo, Helen Harvey, David Henderson Slater, Vijay Kolli, Alex Dickinson

**Affiliations:** 1 Mechanical Engineering Department, University of Southampton, Mailpoint M7, University Road, Highfield, Southampton, SO17 1BJ, United Kingdom, 44 2380595394; 2 Radii Devices Ltd, Bristol, United Kingdom; 3 Portsmouth Hospitals NHS Trust, Portsmouth, United Kingdom; 4 Opcare Ltd, Abingdon, United Kingdom; 5 St George's Hospital, London, United Kingdom; 6 North Bristol NHS Trust, Bristol, United Kingdom; 7 Oxford University Hospitals NHS Trust, Oxford, United Kingdom; 8 Nuffield Department of Orthopaedics and Musculoskeletal Sciences, University of Oxford, Oxford, United Kingdom

**Keywords:** CAD/CAM, prosthetic socket design, transtibial, evidence-based practice, qualitative research, computer aided design and manufacturing

## Abstract

**Background:**

Personalized prosthetic socket design depends upon highly skilled prosthetists. They aim to balance functional human-prosthesis coupling with safe, comfortable load transmission from the prosthesis to the skeleton, through vulnerable skin and soft tissues. Both traditional plaster and computer-aided design and manufacturing (CAD/CAM) methods are iterative, and sharing knowledge is difficult. Evidence-generated (EG) sockets derived from past computer-aided socket design (CASD) records could provide a personalized starting point for limb fitting, potentially reducing time spent on basic design and enabling prosthetists to focus on more highly-skilled customization.

**Objective:**

This study aimed to assess the comfort of EG sockets, generated from past CASD records.

**Methods:**

A crossover trial compared EG sockets, derived from 163 previous transtibial devices, with conventional clinician-led CAD/CAM sockets. Noninferiority was assessed for the socket comfort score (SCS) outcome measure, and semistructured interviews provided in-depth user analysis. The setting was 3 UK National Health Service clinics, with 17 participants with 19 transtibial amputations.

**Results:**

EG sockets had no statistically significant difference in comfort compared with clinician-led control sockets (median SCS 8.6 for EG sockets and 8.8 for CAD/CAM controls; *P*=.43, effect size=0.05), but a lower variability in SCS across the group (95% CIs 8.0‐9.0 for EG and 7.5‐9.5 for CAD/CAM devices, respectively). Analysis of interviews revealed themes around fitting session experiences, similarities, and differences between the EG and CAD/CAM control sockets, and residual limb factors impacting perceptions of socket comfort. These provided insights into the participants’ experience of the study and the value of expert prosthetist input in socket design.

**Conclusions:**

EG sockets demonstrated noninferiority to conventional clinical CASD practice in terms of socket comfort. Both quantitative and qualitative results indicated how clinician input remains essential and is valued by prosthesis users. Work is underway to incorporate the EG sockets into CASD software such that they can act as a digital starting point for modification by expert clinicians at fitting, potentially reducing time spent on basic design, enabling prosthetists to focus on more highly-skilled customization and co-design with their patients.

## Introduction

The lower limb prosthetics community has worked since the 1980s toward computer-aided design and manufacturing (CAD/CAM) technologies to support prosthetic socket design and fabrication workflows [1]. Within CAD/CAM, computer-aided socket design (CASD) is defined as the strategic modification of a 3D digital representation of the residual limb shape in software by an expert human prosthetist, producing a rectified socket shape design. The “CAM” typically refers to fabricating the socket by producing a corresponding rigid foam mold carved using a computer numerical-controlled robotic carver, followed by draping or lamination. CAD/CAM have been described as offering significant potential benefits over conventional plaster of Paris approaches [2], including reduced manual work time and exposure to plaster, an occupational health risk. This could allow clinicians to spend a greater proportion of their time in direct patient interactions, which might enhance patient engagement and facilitate shared decision-making [2]. Early CAD/CAM results were comparable with traditionally produced sockets but took longer and needed more adjustments [1-4]. However, over the learning curve, today clinicians using CAD/CAM can achieve clinical results that are comparable while saving time and delivering a better quality of life outcome [5-8].

These technologies were also proposed to offer opportunities for evidence-based decision support in socket design [1]. CASD generates a perpetual, quantitative design record, whereas the plaster design is destroyed upon socket fabrication. A digital record has value for education, peer support on complex cases, and if implemented for clinical decision support, further time savings to focus on the most highly skilled and value-added parts of socket personalization [3,9]. However, despite proposals since the 1980s to augment CAD/CAM by providing the ability to refer to previous design records [10], it appears that the full potential of the benefits offered through these rich datasets to improve the prosthetic rehabilitation process has not been fully exploited.

In particular, data might be used for generating socket design recommendations, which we define as evidence-generated (EG) sockets. Since Dean and Saunders in 1985 [11], clinical innovators have considered an alternative to performing CASD in a manner analogous to manual work with plaster, whereby a user could apply averaged rectifications from previous designs as an “overlay,” or select from a database of different size and shape “primitive” or “template” sockets, and scale and adjust them to fit to a newly presenting patient’s residual limb shape. This has been demonstrated for transtibial [3,9,12-14] and transfemoral socket designs [15,16], and in orthotics for scoliosis braces [17,18]. Recent publications present alternative methods of informing the socket design process by data, including fuzzy logic or inference to map linguistic descriptions of socket design approaches and patient descriptors, for application to new people [19,20], simulation of load-bearing and optimization for automating transradial socket design [21,22]. However, these studies have only been demonstrated in research settings and have not yet been clinically applied. Anecdotally, today, most CASD software packages in clinical use define templates as structured design workflows or sequences to guide users in applying their own choice of rectifications and gross design features.

The application of such methods would benefit from a systematic study of rectification design practice, although this is limited in the scientific literature [23] since the “Automated Fabrication of Mobility Aids” project [12]. Recently, researchers have begun to leverage high-resolution 3D scans and CASD data to investigate rectification sizes in transtibial and transradial design [24-26], and most recently, probabilistic methods have been used to derive insights into transtibial socket design strategy through the associations between design features [27]. Building upon those insights, this study aims to evaluate an EG socket concept developed by Radii Devices Ltd and the University of Southampton, with UK service provider Opcare Ltd providing data and expert clinical design insight. The objective was to compare the EG socket design approach with clinician-led CASD, using socket comfort outcome measures and capturing the patients’ experiences through qualitative interviews.

## Methods

This study is reported using the STROBE (Strengthening the Reporting of Observational studies in Epidemiology) cross-sectional reporting guidelines [28].

### Patient and Public Involvement and Engagement

The study research question was informed by patient and public involvement and engagement (PPIE) group discussions [29], which highlighted how socket comfort is paramount but difficult to achieve, and that delays between assessment and device provision can impair fitting. PPIE contributors also expressed support for sharing knowledge between prosthetics centers to enable service improvement. Collaboration with the Alex Lewis Trust during the study development reinforced this and provided review and feedback of the study design, recruitment posters, participant information sheets, and consent forms. PPIE contributors are also actively involved in disseminating the study findings to patients.

### Study Design

**Figure 1.**
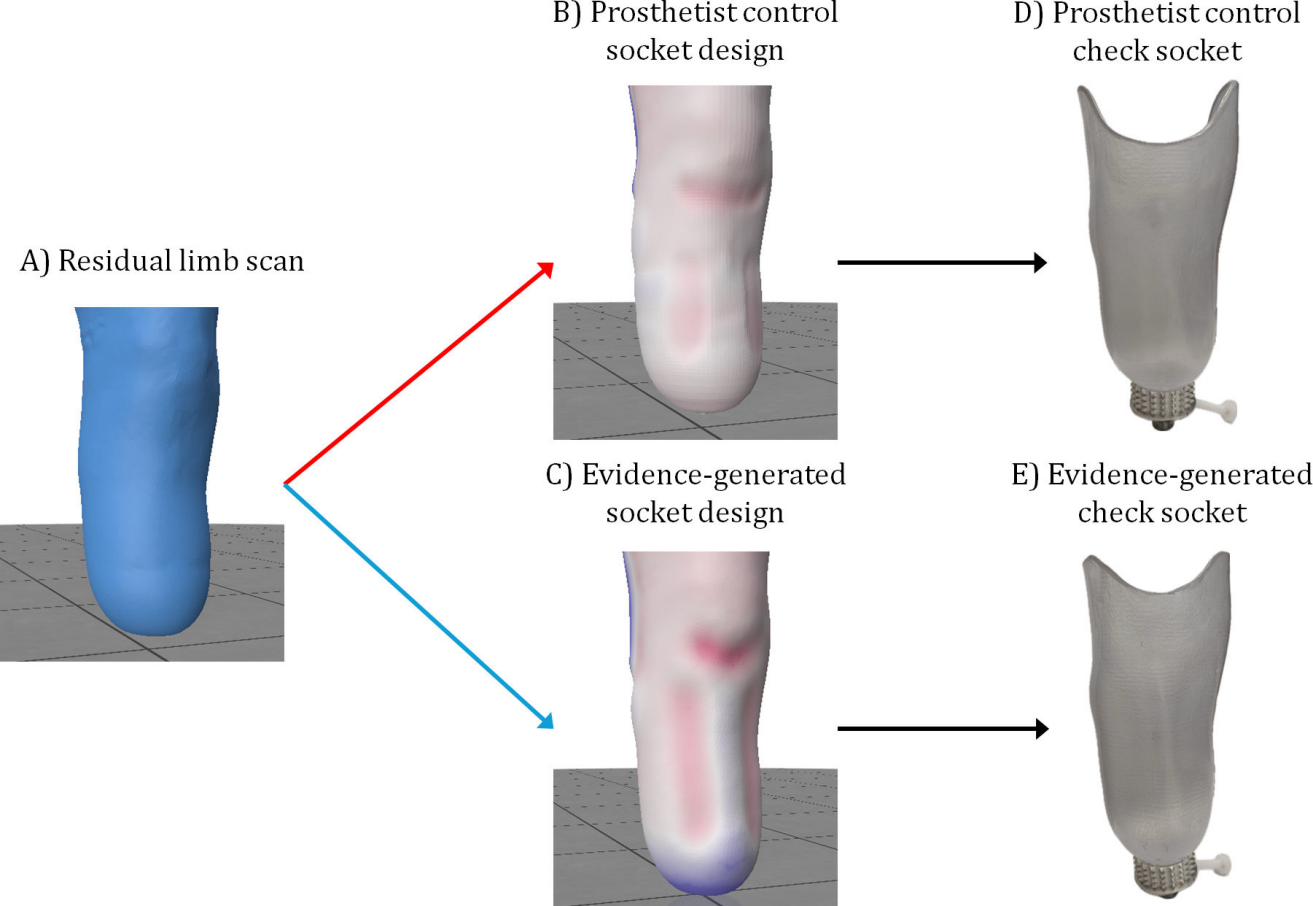
For an exemplar participant: (A) the residual limb scan over a sock; (B) the prosthetist’s socket rectification design; (C) the evidence-generated design before adding brimline; and (D) and (E) the corresponding fitted check sockets. In rectification design maps, the color red represents a carve or press-fit between socket and limb, white represents exact fit, and blue represents buildup or limb-socket gap.

The study used a single-blind, crossover design to assess comfort at socket fitting, followed by a qualitative study of semistructured interviews. An EG transtibial draped thermoplastic check socket was compared with a control CAD/CAM socket, which was designed by a prosthetist using Tracer CASD software (Ohio Willowwood Co) from the same residual limb 3D scan (Figure 1). Participants used the same interface as intended in the definitive device (ie, liners or socks). Quantitative and qualitative data were collected and analyzed independently and interpreted together. This study design was selected to capture the patient’s comparative experience of socket fitting from quantitative and qualitative perspectives, developed from a foundational CAD/CAM versus traditional socket comparison study [5] and with recent precedent in clinical assessment of adjustable sockets [30]. This study design was chosen because, first, new digital design and fabrication technologies are often considered in small scale or low technology readiness level trials in the scientific literature but are often not tested in power-sized, blinded, or controlled trials, with consideration of qualitative service user experience alongside quantitative outcome measures [23]; and second, it is important to understand the patient’s perceived experience. This may relate to socket fit and comfort, for which detailed, open descriptions offer more nuanced insights than a simple socket comfort score (SCS) [31]. It may also relate to the user’s perspective of device design and fabrication, to promote shared decision-making [32] and its potential benefits regarding patient engagement in and understanding of care.

As such, the work aligns with recently published perspectives on ethical considerations for development and clinical translation of prosthetic technologies [33].

### Ethical Considerations

Ethical approval was granted by institutional (ERGO 76033.A3) and UK national review boards (IRAS 313408 and HRAREC 22/YH/0215). Inclusion criteria included people aged 18 years and older, with transtibial amputation, deemed ready for a new prosthetic socket by their prosthetist in their usual clinical pathway, and willing and able to tolerate trialing 2 sockets at a fitting session. Convenience sampling was used to identify participants at 3 UK prosthetic rehabilitation centers. All participants provided written, informed consent (Multimedia Appendix 1); and the presented data are selected to avoid identification. Participants were not compensated.

### Evidence-Generated Socket Design Method

A socket generation method was applied, based upon evidence of expert practice, building upon previous work [27]. To review briefly, a dataset of 163 transtibial residual limb 3D scans and corresponding socket designs was accessed in .aop format (version AAOP1), including labeled landmarks, exported from Omega software (Ohio WillowWood Co) with cylindrical sampling at a maximum 3° spacing on a minimum of 90 slices. These were produced at a single, large UK physical enablement center by 4 prosthetists, all with a minimum BSc qualification, of whom 2 had more than 20 years of experience, one had 5‐10 years, and the other was a first-year graduate. The corresponding patient characteristics, such as gender, age, time since amputation, and reason for amputation, were also collated. A Statistical Shape Model was generated to describe the residual limb shape and size with a minimal number of dimensions, corresponding to principal “modes” of variation. The training datasets were aligned with the assistance of the landmarks, registered, and a principal component analysis was conducted using the full surface shapes of the residual limb scans. Socket design features of local rectifications (patella tendon bar carve, paratibial carves, fibula head build, distal end build, distal tibia build, anterior tibia build, and supracondylar carves) and gross volume change were extracted from the dataset. Bayesian inference was applied to analyze the probabilistic association between patient characteristics and principal modes of limb shape variation (inputs), and the extracted design features for the 163 limb-socket pairs. Finally, the same inputs were obtained for the study participants, and check sockets were created by automatically applying modifications to their landmarked residual limb 3D scans, which were predicted using the statistical model. These were applied to the limb shape scan and saved as a .stl mesh file (output).

### Quantitative Study

The EG socket design was generated automatically except for the brim line, which the fabrication technician was requested to apply in the same location as the control, prosthetist-designed socket. The prosthetist then worked through their standard fitting and assessment procedure for both sockets (Multimedia Appendix 2), including an SCS. At the fitting appointment, the prosthetist chose which socket was trialed first, and the patient participant was blinded to which socket was prosthetist-designed, CAD/CAM, or EG. At the end of the session, the patient participant and their clinician were allowed free choice over which check socket design was to be used for the definitive prosthesis, on the basis of their mutual agreement without any intervention from the researchers.

A power calculation indicated a sample size of 19 was required to test noninferiority in a crossover trial (power=0.9, significance level=0.05, mean difference=0, noninferiority limit=1.21 [34], and population SCS SD 1.2 [24]). A Shapiro-Wilk test indicated the control SCS data were not normally distributed (*P*=.01), so nonparametric descriptive statistics were calculated with CIs using the bootstrap method [35], and the paired Wilcoxon Signed-Rank test was used to assess the statistical significance of the difference between the sockets.

### Qualitative Study

Following the quantitative study, 2 semistructured interviews were carried out to capture participants’ views and experiences of in-clinic fitting of 2 sockets designed in different ways, and general usability of their new prosthetic socket. The first was immediately after completion of the socket fitting, and the second was 1 month afterwards (Multimedia Appendix 3). Interviews were audio-recorded, anonymized, and transcribed verbatim by a professional transcribing company. A member of the research team (FM) checked a sample of transcripts against the audio recordings for accuracy. The transcripts were analyzed using thematic analysis, which provided a flexible approach to ascertain a clear understanding of the comparability of socket comfort and views of the processes [36]. The thematic analysis approach consisted of (1) familiarization with the data, (2) coding by hand and using NVivo software (Lumivero), (3) generating initial themes, and (4) reviewing and finalizing the final themes to capture consistent patterns within the data and key meaning relevant to the research questions [36]. One team member (MD-H) led all stages of the analysis; 2 other members (FM and JB) coded different sections of the data and together agreed on the final themes, providing verification and allowing for a range of interpretations.

## Results

### Recruitment

**Figure 2.**
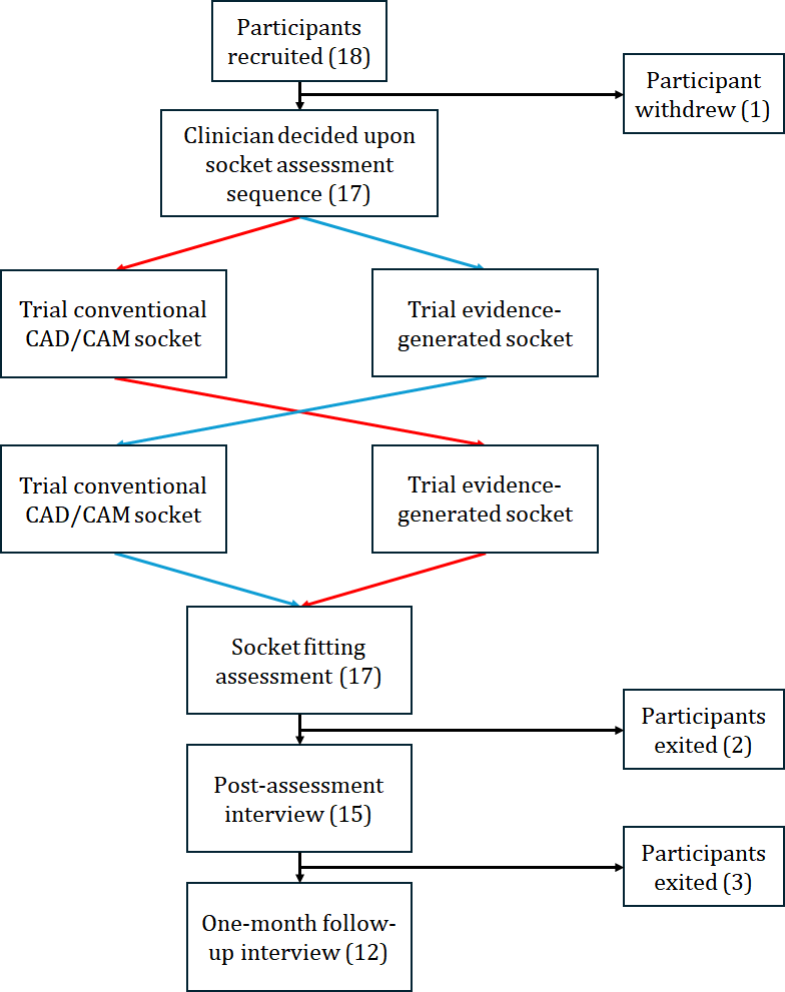
Study design and recruitment diagram. CAD/CAM: computer-aided design and manufacturing.

In total, 17 recruited participants with 19 residual limbs completed the socket assessment study between March and November 2023 (Table 1 and Figure 2). Furthermore, 6 prosthetists designed control sockets for between 1 and 9 participants. Approval was initially granted to compare transparent check sockets, but due to slow recruitment, a study protocol amendment was approved to compare the EG check socket to a definitive CAD/CAM prosthetist-designed socket, reflecting more common practice in the participating clinics.

In addition, 2 participants exited before completing the postassessment interview, and a further 3 were not contactable for the 1-month follow-up interview.

**Table 1.** Description of participants.

Characteristic	Statistical value
Side (n=19), n	
Left	8
Right	11
Sex (n=17), n	
Women	1
Men	16
Age (years; n=17), median (IQR)	66 (51‐73)
Time since amputation (years; n=19), median (IQR)	4.0 (2.5‐8.9)
Reason for amputation (n=19), n	
Infection or diabetic foot	6
Dysvascularity	4
Trauma	3
Ischemia or CLTI^a^	3
Sepsis	1
Peripheral Neuropathy	1
Chronic osteomyelitis secondary to trauma	1
Reasons for new socket (n=19), n	
Socket too large or limb shrinkage	12
Requested new suspension method	3
Socket too small or tight	2
Alignment incorrect	1
N/R^b^	3
Participant activity level (n=17)	
A1: In-house walking or transfers only	1
A2: Walking on flat ground only	6
A3: Normal everyday walking	8
A4: Additional high impact or energy activities	0
N/R	2

a CLTI: critical limb-threatening ischemia; activity level as assessed by the participants’ normal clinical team.

b N/R: not reported.

### Quantitative Study Findings

**Figure 3.**
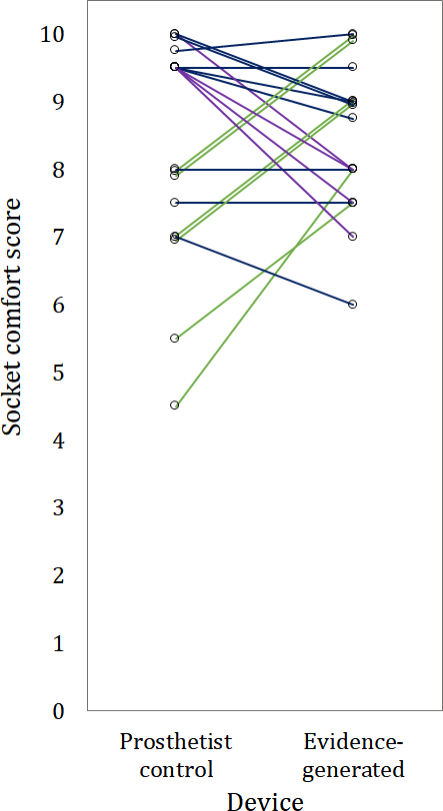
Noninferiority crossover study results comparing socket comfort score between the prosthetist control and evidence-generated sockets, for 19 fittings across 17 participants, presented as a paired comparison chart. Color coding denotes higher (green), the same (blue), or lower (purple) socket comfort score for evidence-generated than prosthetist control devices, within the noninferiority threshold of 1.21 points.

The unmodified EG sockets had no statistically significant difference in SCS compared with prosthetist-designed CAD/CAM control sockets (median SCS 8.6 for EG sockets and 8.8 for CAD/CAM controls; *P*=.43 and effect size=0.05 in the paired significance test, Figure 3). Lower variability in SCS was observed across the study group for the EG sockets than the control sockets (95% CIs 8.0‐9.0 and 7.5‐9.5, respectively). Of the 19 EG sockets, 9 were given a comfort score within the noninferiority limit (standard error of measurement 1.21 points [34]) of the control. In total, 6 EG sockets were rated as more comfortable than the CAD/CAM control, by between 2.0 and 3.5 points; 4 EG sockets demonstrated lower comfort than the CAD/CAM control, by 1.5 to 2.5 points.

### Qualitative Study Findings

Due to the significant impact and diverse experiences related to socket comfort, thematic analysis identified several broader themes that were beyond this paper’s objective. Focus is therefore limited to specific themes that offer insights into the participants’ views and experiences relating to the sockets produced by the 2 design approaches (Table 2).

**Table 2.** Themes identified in semistructured interviews relating to comparison between comfort of the fitted sockets and reflections on the socket fitting process.

Theme	Description
Theme 1	Experiences of the comparative fitting session
Theme 2	Similarity between the compared sockets
Theme 3	Differences between the compared sockets
Theme 4	Residual limb factors impacting perceptions of socket comfort

#### Theme 1: Experiences of the Comparative Fitting Session

Both devices were fabricated before the fitting session, and when discussing the session, participants reflected on several aspects that they felt impacted their experiences. Several participants described feeling that it went “*well*,” “*fine*,” or “*ok*,” which illustrated that a process involving 2 different sockets was not a particular issue. For example, 1 participant discussed their experience in terms of the output of the session:

*I got something out of it, it looks like this time if you know what I mean. At the end of this fitting there was something that looked like it was ready for me*.

[Participant 10, male, 58 y, amputation following trauma, gel cushion liner]

Other participants discussed how they felt about comparing the 2 sockets and commented on how they did not know who designed either of the 2 sockets, for example:

*I didn’t know who designed and who produced each socket*.

[Participant 1, male, 81 y, infection, sock]

Other participants discussed their experience in terms of their expectations of addressing their specific socket fit issues relating to their residual limb. For example:

*The only thing I’ve got hope for there is that the bottom of my stump shrinks a lot more before it gets thinner and thinner because that’s the only thing that’s stopping me having a smaller prosthetic is that stump. It’s where they added all the bits on*.

[Participant 10, male, 58 y, amputation following trauma, gel cushion liner]

#### Theme 2: Similarity Between the Compared Sockets

Several participants described not feeling any difference in the level of comfort between the sockets, regardless of which design process was used. For some participants, this appeared to be related to feeling that both sockets were equally comfortable, with descriptions of how they found it difficult to decide if one was more comfortable than the other:

*As far as comfort there was very little difference in it really to be honest as far as I could judge at the time*.

[Participant 19, male, 71 y, amputation due to ischemia, gel liner]

Some participants compared the comfort of the two new sockets to their previous device. For example:

*I feel a lot more comfortable really than the old one*.

[Participant 1, male, 81 y, infection, sock]

This similarity in the level of comfort between the 2 new sockets was described by some participants in relation to both sockets feeling equally “*firm*,” “*stable*,” and “*both fitting well*,” and both sockets feeling very “*natural*,” as described by 1 participant:

*Absolutely fine. They were both very, very close to being completely natural.*

[Participant 4, male, 30 y, elective amputation following trauma, suction liner]

However, for other participants, the similarity between the sockets appeared to be associated with feeling similar levels of pain:

...*walking with them as I was walking there was the same amount of pain*...

[Participant 11, female, 84 y, peripheral vascular disease, cushion liner]

#### Theme 3: Differences Between the Compared Sockets

Other participants noticed significant differences in the comfort of the 2 sockets, from positive and negative perspectives. The reported comparative fitment assessments were associated with some of the factors the participants raised in theme 2, around similarities in fit, but also included differing, design-related factors such as the height of the socket, local shape details associated with bony prominences, and dynamic fit. Participants mentioned issues such as pressure points, discomfort at the back of the leg, and feelings of tightness. For example:

*One felt really tight. I like it tight when I get a new socket because it lasts longer. One felt a lot more comfortable at the front where my bone is closer to the skin*.

[Participant 12, male, 40 y, amputation due to chronic ulcers, seal-in liner]

Differences in the size of the sockets also affected comfort. One participant explained:

*The taller one was a bit more uncomfortable due to how high it was. When you bend your leg down, it hits the back of your leg*.

[Participant 4, male, 30 y, elective amputation post trauma, suction liner]

Some participants felt that the height of the socket made it feel more natural. These differences impacted participants’ walking, with 1 socket feeling easier to walk in than the other

*One socket was absolutely perfect. The other one had different movement, slightly different, but it seemed like you walked quicker with it. It was a nice, easy movement*.

[Participant 8, male, 76 y, ischemic amputation, pin liner]

#### Theme 4: Residual Limb Factors Impacting Perceptions of Socket Comfort

While comparing the 2 sockets, participants discussed sensitive areas that affected their comfort, such as spots on the crest of the shin and other specific sore areas. These issues were often ongoing and considered by the prosthetist during socket design:

*I’ve always had this issue. We’ve had to shave out a bit of the socket to ease the pressure. But I think it’s just the shape of my stump, and it’s something that will always be an issue and I’m always aware of it*.

[Participant 13, male, 32 y, elective amputation post trauma, no interface details recorded]

Some participants reflected on the adjustment after the fitting process, saying:

*I don’t know if the second one on the right whether it needed any more adjustment. Maybe a little bit but I would say they are fairly comfortable.*

[Participant 16/17, male, 70 y, diabetic amputations, no interface details recorded]

Other factors, also not necessarily directly related to the design process, appeared to impact the participants’ views of which of the 2 new sockets were more comfortable. For example, 1 participant described how their residual limb had shrunk since their last prosthetic fitting, and this led to the new socket feeling more comfortable:

*But it does feel better and that clear one that I had in my old socket … had more movement because the stump had shrunk so much. So, it’s just a case of getting used to having this because it’s slightly smaller and it’s hugging the stump. Perhaps where I had the freedom before where it moves about a lot more … I’ve got to get used to the new feelings of it*.

[Participant 6, male, 51 y, dysvascular amputation, no interface devices]

## Discussion

### Principal Results

This study demonstrated the noninferiority of the EG socket design method in comparison with conventional clinician-led CAD/CAM socket design at initial fitting. SCS indicated similar comfort on average and reduced variability across the cohort for the EG sockets. Participant interviews confirmed this assessment and added a more detailed understanding of socket satisfaction.

Thematic analysis also revealed the patients’ detailed understanding of the nuances of their prosthesis design and its fit and demonstrated the importance of ensuring a trained, experienced clinician directs the application of technology-enhanced socket design processes. The comfort results indicated noninferiority of the EG sockets’ fundamental design without any personalized clinician input. However, the qualitative study provided evidence to support clinical usage of EG sockets with an expert human in the loop to make design decisions in response to patients’ individual and complex needs [12,24,37]. That might include local design modifications in response to vulnerable sites on that individual’s residual limb or accommodating the patient’s preferences for tightness of fit. This was identified for the single participant whose EG socket was rated with SCS below 7 (participant 16/17, male, 70 y, diabetic amputation), for whom the score of 7 was attributed to an unusual tissue sensitivity at a supracondylar site, where the CAD/CAM control socket featured a local modification. The same point may explain the observed trend where all 4 EG sockets that were rated as less comfortable than the CAD/CAM control were cases where the control scored an SCS of 9 to 10, where such personalization has evidently been successful. Patient-specific local areas of sensitivity and corresponding design changes inherently cannot be generalized, so this justifies the importance of an expert in the design process, who retains clinical responsibility.

### Comparison With Previous Work

The complementary evidence provided by this multiple methods approach demonstrates the value of exploring the participants’ experience more deeply through interviewing, to enhance what can be learned from objective measurement [38]. However, this approach is somewhat unusual in the assessment of prosthetic technologies [30]. The study involved a large interdisciplinary team, which enabled separation of trial socket design, clinical assessment, interviewing, data analysis, and interpretation, in an attempt to minimize potential researcher biases. The combined synthesis of the SCS and interviews revealed the participants’ detailed understanding of the influence of residual limb shape and corresponding socket design upon their comfort and function, illustrative of the value of excellent prosthetist-patient communication. These technologies may offer an opportunity to improve patient experience through understanding of their care [39] or even shared decision-making [40]. The use of an EG socket design process may enable greater focus on the higher value-added aspects of personalization and might be easier to perform in front of the patient [24]. Most notably, this observation justifies ongoing work with clinician stakeholders, for example, to indicate specific areas in which human input is particularly important for fitting EG sockets to newly presenting individuals, and in the development of software interfaces, building upon methods reported by Ngan et al [41].

### Limitations

The study included a relatively diverse group of patients accessing prosthetic rehabilitation services, except for gender, where the recruited participants were predominantly men (16/17) [42], which may impact the study’s generalizability. Women are less likely to have a major amputation and to be successfully fitted with a prosthesis [43], enter prosthetic rehabilitation later [44], and are underrepresented in research cohorts [45]. It is not clear why this study’s convenience sampling resulted in an imbalance, but this illustrates the need to ensure that further studies include more diverse gender representation, as the experiences of men and women may be different. However, the distribution of randomly sampled people whose socket designs were used to train the socket design evidence model [27] was representative of the population of people with transtibial amputations, and external validity is supported by their diverse range of ages, reasons for limb absence, activity levels, and time since amputation. Further bias may arise from the recruited participants’ age profile (median 66, IQR 51‐73, range 30‐84 y), which was similar on average but narrower in range than the general population of people using prosthetic limbs [42]. The participants' age profile was older on average but similar in range to the historic socket design dataset (median 60, IQR 50‐71, range 20‐94 y [27]). Finally, the training datasets came from clinicians at a single center, whose practice may be similar, potentially limiting the diversity of design approaches.

The study also has limitations in its single timepoint and outcome measure, where previous studies comparing CAD/CAM with plaster design and fabrication considered quality of life [7] and more extensive assessments of comfort, fit, cosmesis, weight, and function alongside clinical workload and productivity measures [9]. The study cannot indicate how socket comfort would develop long-term, and it may be preferable to use the expanded SCS for best, worst, and average comfort over a longer period [46]. However, this study replicates the clinical assessment of trial and definitive sockets in UK National Health Service settings and provides additional insight since participants could compare 2 socket options side-by-side and select their preference, with interviews proceeding a month after fitting. A different comparison, versus plaster socket design methods, might provide additional insights, but the study was designed to compare with conventional CAD/CAM as the standard of care in the participating centers, and indeed, the prevalence of CAD/CAM is rising. Finally, the study was not formally randomized, and prosthetists generally fitted their own design socket first, meaning a carryover effect cannot be excluded. Alongside this limitation, single-blinding could not apply to 3 participants for whom the EG check socket was compared with a definitive CAD/CAM control socket, instead of comparing 2 visually-similar check sockets.

### Clinical Messages

First, EG transtibial prosthetic socket designs compared well with devices produced by conventional CAD/CAM clinical practice, in terms of comfort at socket fitting and patient feedback in semistructured interviews.

Second, qualitative feedback confirmed that clinician input remains essential to incorporate patient-specific socket design details in response to local sites of sensitivity or tissue vulnerability, or preference: details that cannot be generalized.

Finally, work is underway to incorporate EG socket designs into CASD software such that they can be modified at fitting, enhancing evidence-based practice as a support tool to help qualified clinicians leverage their experience and skill and enable co-design with the prosthesis user.

### Conclusions

Overall, the study findings support wider clinical use of EG transtibial sockets, but also demonstrate the importance of delivering this technology in a way that facilitates prosthetist input in tailoring the design to their individual patients’ needs and provides maximal opportunity for their communication. Such an implementation may enable prosthetists to take the advantages offered by technology while retaining or ideally enhancing trust through human-centered prosthetic rehabilitation.

## Supplementary material

10.2196/69962 Multimedia Appendix 1 Participant consent form.

10.2196/69962 Multimedia Appendix 2 Transtibial checkout procedure.

10.2196/69962 Multimedia Appendix 3 Semistructured interview schedules.

## Abbreviations

CAD/CAM: computer-aided design and manufacturing
CASD: computer-aided socket design
EG: evidence-generated
PPIE: patient and participant involvement and engagement
SCS: socket comfort score
STROBE: Strengthening the Reporting of Observational Studies in Epidemiology
