# Supplementary material for: Evidence-Generated Sockets for Transtibial Prosthetic Limbs Compared With Conventional Computer-Aided Designs: A Multiple-Methods Study From the Patient’s Perspective
Source: JMIR Rehabil Assist Technol. 2025 Aug 21;12:e69962. doi: 10.2196/69962 (PMC12370269; doi:10.2196/69962)
Supplement: Multimedia Appendix 1 [file rehab-v12-e69962-s001.docx]

**PARTICIPANT CONSENT FORM**

**Title of Study: Comparing sockets designed by Radii Devices software with those designed by clinician**

**REC Reference: IRAS Project ID: 313408 ERGO NO: 76033**

**Stage One**

If you wish to take part in **Stage One** of this study, **please place your initials in each of the boxes below, sign and date this form.** Please initial box

| 1. I confirm that I have read and I understand the information sheet **version 1** dated **01/07/22** for the above study and have had the opportunity to ask any questions about the study and any questions have been answered to my satisfaction.   ***Initials*** | | | | | | | | | | | | | | | |
| --- | --- | --- | --- | --- | --- | --- | --- | --- | --- | --- | --- | --- | --- | --- | --- |
| 1. I understand that my participation is voluntary and that I am free to withdraw at any time without giving any reason, and without my medical care or legal rights being affected.   ***Initials***   1. I understand that relevant sections of my medical notes and data collected during the study, may be looked at by individuals from University of Southampton, North Bristol NHS Trust and Radii Devices where it is relevant to my taking part in this research. I give permission for these individuals to have access to my records.   ***Initials***   1. I agree to North Bristol NHS Trust and the University of Southampton holding copies of my consent form and other study related documents to assist with study data collection.   ***Initials***   1. In the event that I lose the ability to consent during the study, I understand that I will be withdrawn from the study but that data already collected with consent would be retained and used in the study. No further data would be collected.   ***Initials***  ***Initials***   1. I am willing to trial two sockets at my fitting appointment. 2. **I agree to take part in this study.**   ***Initials***  Now, please turn over  **Stage One- Please sign and date this form below:**   \|  \| \|  \| *d* \| \| *d* \| \| */* \| \| *m* \| \| *m* \| \| */* \| \| *y* \| \| *y* \| \| *y* \| \| *y* \| \|  \| \|  \| \| --- \| --- \| --- \| --- \| --- \| --- \| --- \| --- \| --- \| --- \| --- \| --- \| --- \| --- \| --- \| --- \| --- \| --- \| --- \| --- \| --- \| --- \| --- \| --- \| --- \| --- \| \| Print name \|  \| \| \|  \| \|  \| \| / \|  \|  \| \| / \| \| 2 \| \| 0 \| \|  \| \|  \| \|  \| \| Signature \| \| \| \| Name of participant (*please print*) \| \| \| \| Date \| \| \| \| \| \| \| \| \| \| \| \| \| \| \| \| \| \| Signature of participant \| \| \| \| \| \|  \| \| \| \| *d* \| \| *d* \| \| */* \| *m* \| *m* \| \| */* \| \| *y* \| \| *y* \| \| *y* \| \| *y* \| \|  \| \| \| \| \| \| Print name \|  \| \| \|  \| \|  \| \| / \|  \|  \| \| / \| \| 2 \| \| 0 \| \|  \| \|  \| \|  \| \| Signature \| \| \| \| Name of person taking consent  (*please print*) \| \| \| \| Date \| \| \| \| \| \| \| \| \| \| \| \| \| \| \| \| \| \| Signature of person taking consent \| \| \| \| \|   **Stage Two (additional optional extra)**  **The following are not essential for you participation in Stage One.** If you wish to take part in **Stage Two** of this study, **please place your initials in each of the boxes below.**  ***Initials***   1. I am willing to have two audio-recorded interviews to share my socket fitting experiences 2. I understand that anonymised written quotations from the interview may be used in  publications and presentations.   ***Initials***   1. I give permission for the researchers to publish anonymised direct quotations for the purpose of sharing research.   ***Initials***  ***Initials***   1. I understand that the information collected about me will be used to support other research in the future, and may be shared anonymously with other researchers.   *No*  *Yes*   \| 1. Researchers from this study would like to contact people who agree to take part in this study to see if they would be interested in helping with other related studies. If you would like to be sent information about related studies, please initial in the Yes or No box.   ***Initials***  ***Initials***  **Stage Two- Please sign and date this form below:** \| \| --- \| | | | | | | | | | | | | | | | |
| Print name |  |  |  | / |  |  | / | 2 | 0 |  |  |  | Signature |  |  |
| Name of participant (*please print*) | | Date | | | | | | | | | | Signature of participant | |  |  |
| Print name |  |  |  | / |  |  | / | 2 | 0 |  |  |  | Signature |  |  |
| Name of person taking consent  (*please print*) | | Date | | | | | | | | | | Signature of person taking consent | |  |  |

**[Original to be kept in Site File; one copy given to participant (upon request); one copy in participant’s records; one copy to be returned to the University of Southampton]**
