# Supplementary material for: Evidence-Generated Sockets for Transtibial Prosthetic Limbs Compared With Conventional Computer-Aided Designs: A Multiple-Methods Study From the Patient’s Perspective
Source: JMIR Rehabil Assist Technol. 2025 Aug 21;12:e69962. doi: 10.2196/69962 (PMC12370269; doi:10.2196/69962)
Supplement: Multimedia Appendix 2 [file rehab-v12-e69962-s002.docx]

***Transtibial Checkout Procedure***

***Patient sitting***

| Can the patient don the prosthesis without assistance? |
| --- |
| Is the socket in the correct transverse position i.e. inferior border of patella is congruent with anterior trimline? |
| Does the patient report discomfort, tightness, pins and needles etc? |
| Is the PTB in line with the mid patella tendon when distal force is applied? |
| Is the suspension method appropriately positioned and sufficiently secure to suspend the prosthesis? |
| Can patient flex the knee to an acceptable extent? |
| Does the trimline protrude above the level of the thigh? (consider suspension method) |

***Patient standing***

| Is the socket comfortable? Consider use of socket comfort score. |
| --- |
| Is length correct? Use ASIS, Iliac crest, PSIS and consider previous pelvic damage. |
| Is the base of gait secure? Consider A/P and M/L of plantar surface of foot. |
| Is suspension satisfactory when prosthesis is lifted vertically? Minor pistoning is inevitable. |
| Are the mediolateral aspects of the socket continuous with proximal anatomy? |
| Does patient report balance as neutral? |
| Is toe out angle correct? |
| Is the shape and profile acceptable to the patient? Primary patients will have oedema which will compromise shape. Excessive contractures will also compromise profile. |

***Patient walking***

| Does the socket remain comfortable? Consider number of socks. |
| --- |
| Is suspension method resulting in minimum pistoning? |
| Are there any gait deviations? Not an exhaustive list:  Mediolateral knee movement  Hyperextension  Foot slap  Excessive or rapid flexion  Lateral trunk bending  Heel whip  Uneven stance/swing ratio  Toe clearance |
| Are there any noises? |

***Following patient walking***

| Can patient doff prosthesis? |
| --- |
| Is marking on patients stump of acceptable intensity and in pressure tolerant areas? |
| Are there any areas of abrasion or open sores? |
| Is the stump discoloured? Consider distal soft tissue. |
