# Supplementary material for: Evidence-Generated Sockets for Transtibial Prosthetic Limbs Compared With Conventional Computer-Aided Designs: A Multiple-Methods Study From the Patient’s Perspective
Source: JMIR Rehabil Assist Technol. 2025 Aug 21;12:e69962. doi: 10.2196/69962 (PMC12370269; doi:10.2196/69962)
Supplement: Multimedia Appendix 3 [file rehab-v12-e69962-s003.docx]

# Semi-Structured Interview Schedules

## Post-fitting session Group A (participants with limb loss)

**Introduction:**

- - *Briefly explain aims of study*
  - *Briefly explain purpose of interview to understand their views and experience of being fitted with the two sockets designed in different ways*
  - *Interested in their own views and experiences*
  - *There are no right and wrong answers*
  - *Explain what will be done with the data collected*
  - *Explain reason for blinding and ask whether they know which socket was designed by Radii*
  - *Have they got any questions?*

**Semi-structured interview questions:**

- How did you find/can you tell me about your experience of today’s socket fitting session?
  - What do you think went?
  - What didn’t go well?
- How did the two sockets you tried on feel?
  - Similar/different?
  - Were certain aspects comfortable/uncomfortable
  - Could you show me on a limb image where?
  - How stable did the sockets feel? Any differences?
  - How Confident would you feel using the socket? Was there any difference between the two regarding how confident you felt?
  - How well do you feel you would be able to do the activities you want? Any differences between the sockets?
- Can you tell me about you overall experience of getting/using a socket so far?
  - Expectations
  - Goals
  - Positives/frustrations
  - Liners and socks used?
  - Do you have any areas of no sensation on your residual limb?
  - Do you have any areas that often feel uncomfortable/comfortable?
  - Do you do anything to make your socket more comfortable or functional to use?
- Is there anything else you would like to share on socket comfort and fit?

## Post-fitting session Group B (Prosthetists)

**Introduction:**

- - *Briefly explain aims of study*
  - *Briefly explain purpose of interview to understand their views and experience of the different design methodologies and fitting the two sockets designed in different ways*
  - *Interested in their own views and experiences*
  - *There are no right and wrong answers*
  - *Explain what will be done with the data collected*
  - *Explain reason for blinding and ask whether they know which socket was designed by Radii*
  - *Have they got any questions?*

**Semi-structured interview questions:**

- How did you find/can you tell me about your experience of today’s socket fitting session?
  - What do you think went well?
  - What didn’t go well?
- What are your thoughts on the two sockets that you fitted?
  - Differences/similarities?
  - Were you able to tell which socket was designed by a prosthetist, and how?
- What do you think would be the benefits/concerns for prosthetists of using this software in-clinic?
- What factors do you think need to be considered to make this useable in clinic?
- Is there anything else that you’d like to share regarding use of a platform including AI to support evidence-based socket design?
- Have your views changed while participating in this study? (if end of study interview)
- Is there anything else you would like to tell us about your experiences with socket design and fit?

## End of study telephone interview Group A (participants with limb loss)

**Introduction:**

- - *Briefly explain aims of study*
  - *Briefly explain purpose of interview to understand their views and experience of using their new socket*
  - *Interested in their own views and experiences*
  - *There are no right and wrong answers*
  - *Explain what will be done with the data collected*
  - *Explain reason for blinding and ask whether they know which socket was designed by Radii*
  - *Have they got any questions?*

**Semi-structured interview questions:**

- How have you found completing the socket comfort diary?
  - What do you think went well?
  - What could have been better? Different methods, more/less reminders? More structured? Number of questions?
- How did you find/can you tell me about your experiences using your new socket over the last month?
  - What went well?
  - What didn’t go well?
  - Have you been back to clinic/contacted your prosthetist?
  - Have you had to manage your fit and comfort yourself over the past month?
  - What kinds of things have you done to make yourself feel more comfortable in the socket/make your socket easier to use?
  - Have these approaches worked?
  - How has the comfort and fit of the socket impacted your daily life?
  - How has the comfort and fit of the socket impacted your quality of life?
  - Goals
  - Expectations
  - Positives/frustrations
- Is there anything else you would like to share on socket comfort and fit?
